# Impact of disease cyclicity on quality-of-life impairment—A mixed method explanatory study

**DOI:** 10.1016/j.jdin.2024.09.007

**Published:** 2024-11-09

**Authors:** Ying Shan Cheung, Winston Tham Zhi Wen, Ashvin R. Chundayil, Phillip Phan, Ellie Choi Ci-En

**Affiliations:** a Division of Dermatology, Department of Medicine, National University Healthcare System, Singapore; b School of Medicine, University College London (UCL), London, United Kingdom; c Department of Medicine, Yong Loo Lin School of Medicine, National University of Singapore, Singapore; d Carey Business School, John Hopkins University, Baltimore, Maryland

**Keywords:** disease cyclicity, eczema, mixed method, psoriasis, qualitative study, symptom burden

## Abstract

**Background:**

Disease cyclicity, a composite measure of unpredictable and daily fluctuations of symptoms, strongly predicts quality-of-life (QoL) impairment.

**Objective:**

To explore the mechanisms by which cyclicity impacts QoL.

**Methods:**

1:1 semi-structured interviews were conducted and qualitatively analyzed using grounded theory. Common themes were identified and used to derive a theoretical framework.

**Results:**

Twenty-three adults, (median age 28.5, range 20-72) with a range of chronic inflammatory diseases including eczema, psoriasis, and inflammatory arthritis were prospectively recruited. Cyclicity, characterized by unpredictability, uncontrollability, and lability, contributed to a varying productivity and inconsistent ability in performing daily activities. Challenges intensified if external expectations of the patient were inconsistent with their fluctuating ability. Coping abilities, shaped by internal and external factors, moderated the relationship between cyclicity and QoL impairment.

**Limitations:**

Qualitative interviews assume patients have accurate insights into their own behaviors. The generalizability of findings may be limited in other populations.

**Conclusion:**

Disease cyclicity defines many inflammatory diseases. This study provides a theoretical framework for understanding and managing the challenges patients with a cyclical condition face.

**Capsule Summary**

• Disease cyclicity leads to inconsistent abilities and unmet expectations due to its lability, unpredictability and uncontrollability.
• Treatment should mitigate disease fluctuations, while coping methods must encompass strategies to manage cyclical patterns.

## Introduction

Quality-of-life (QoL) impairment due to disease is linked to factors like disease severity, social support, and resilience.^1^, ^2^, ^3^ Our recent study of 1053 dermatological patients with eczema or psoriasis identified disease cyclicity as a novel predictor of QoL impairment (standardized B 0.47, SE 0.05, *P* < .001),^4^ exceeding the effect of objective severity, resilience, and personality. This relationship remained significant even after adjusting for disease severity and other characteristics.

Disease cyclicity is a construct measured by the IPQ-R (Illness Perception Questionnaire-Revised), a widely used self-reporting questionnaire of patient’s perceptions of their illnesses.^5^ The questionnaire assesses cyclical timeline perceptions and the control patients’ feel over their illnesses. Cyclicity describes the pattern of biochemical or clinical peak followed by remission causing unpredictable and daily fluctuations of symptoms. It is present in most chronic inflammatory conditions. Current literature on cyclicity reports on QoL during periods of flares and remission,^6^^,^^7^ with a higher QoL impairment in patients with greater disease activity and more frequent flares. There is, however, a lack of literature defining the characteristics of cyclical conditions and why it carries such a large impact on QoL. Uncovering the nuanced reasons why patients with higher disease cyclicity experience greater impairment will provide clues for mitigating disease burden in patients with chronic inflammatory conditions.

## Methods

This sequential mixed methods explanatory study recruited participants from National University Hospital, a tertiary academic dermatological center in Singapore, and through word-of-mouth. Inclusion criteria included being aged 21 and above and having a chronic (>3 months duration) inflammatory disease.

One-on-one in-depth semi-structured interviews were conducted in-person or over videoconferencing in English. The audio was recorded with permission of the participant and later transcribed verbatim for analysis. Interviews were conducted by the authors. Interviewers were trained in gathering qualitative information. Observers, which included medical students and dermatology consultants, could participate and ask questions.

The interviews explored how participants defined cyclicity, the emotions experienced with each phase of the cycle, and how these impacted their personal, social, work, and health care interactions. Initial interview guides (Supplementary File 1, available via Mendeley at https://data.mendeley.com/datasets/r2dw9h9c2p/1) were less structured and became more focused (Supplementary File 2, available via Mendeley at https://data.mendeley.com/datasets/r2dw9h9c2p/1) as themes emerged from each round of interviews. Initial interview durations ranged from 30 to 70 minutes that became progressively shorter as the questions became more focused.

We employed grounded theory^8^ to the interview data to elicit insights. E.C. and C.Y.S. performed line-by-line, followed by analytical focused coding, with team discussions after every 4-5 interviews. Following this, the interview guide was updated to reflect new learnings, while new participants were recruited using theoretical sampling. This cycle of interviewing, coding, and analysis continued until theoretical saturation, defined as a stage when no new information emerges, was reached. Through axial coding, similar codes were grouped into higher-order themes. The team then developed and refined a theoretical framework, which all members unanimously endorsed. The study design follows the Consolidated Criteria for Reporting Qualitative Research (COREQ) guidelines.^9^ (Supplementary File 3, available via Mendeley at https://data.mendeley.com/datasets/r2dw9h9c2p/1). The study was approved by the National Healthcare Group’s Domain Specific Review Board (reference 2021/00110).

## Results

### Participants

Twenty-three participants with various chronic inflammatory diseases were recruited between June 2023 and January 2024. The mean age was 34.1 (SD: 13.7) years. Fifteen (73.9%) were males and 8 (34.7%) were females. Inflammatory conditions included eczema, psoriasis, ankylosing spondylitis, and rheumatoid arthritis, with a mean duration of disease of 16.8 (SD: 11.0) years (Table I).

**Table I:** Demographics of patients interviewed (*n* = 23)

Variable	Number (%)
Age	
Mean (SD)	34.1 (13.7)
Median (range)	28.5 (20-72)
Sex	
Male	15 (65.2%)
Female	8 (34.7%)
Disease	
Eczema	17 (73.9%)
Psoriasis	2 (8.7%)
Urticaria	1 (4.3%)
Rheumatoid arthritis	1 (4.3%)
Ankylosing spondylitis	1 (4.3%)
Osteoarthritis due to hip dysplasia	1 (4.3%)
Duration of disease (y)	
Mean (SD)	16.8 (11.0)
Median (range)	20 (0.5-40)
Interviews	
In person	8 (34.7%)
Over Zoom	15 (65.2%)

### Overall framework

**Fig 1:**
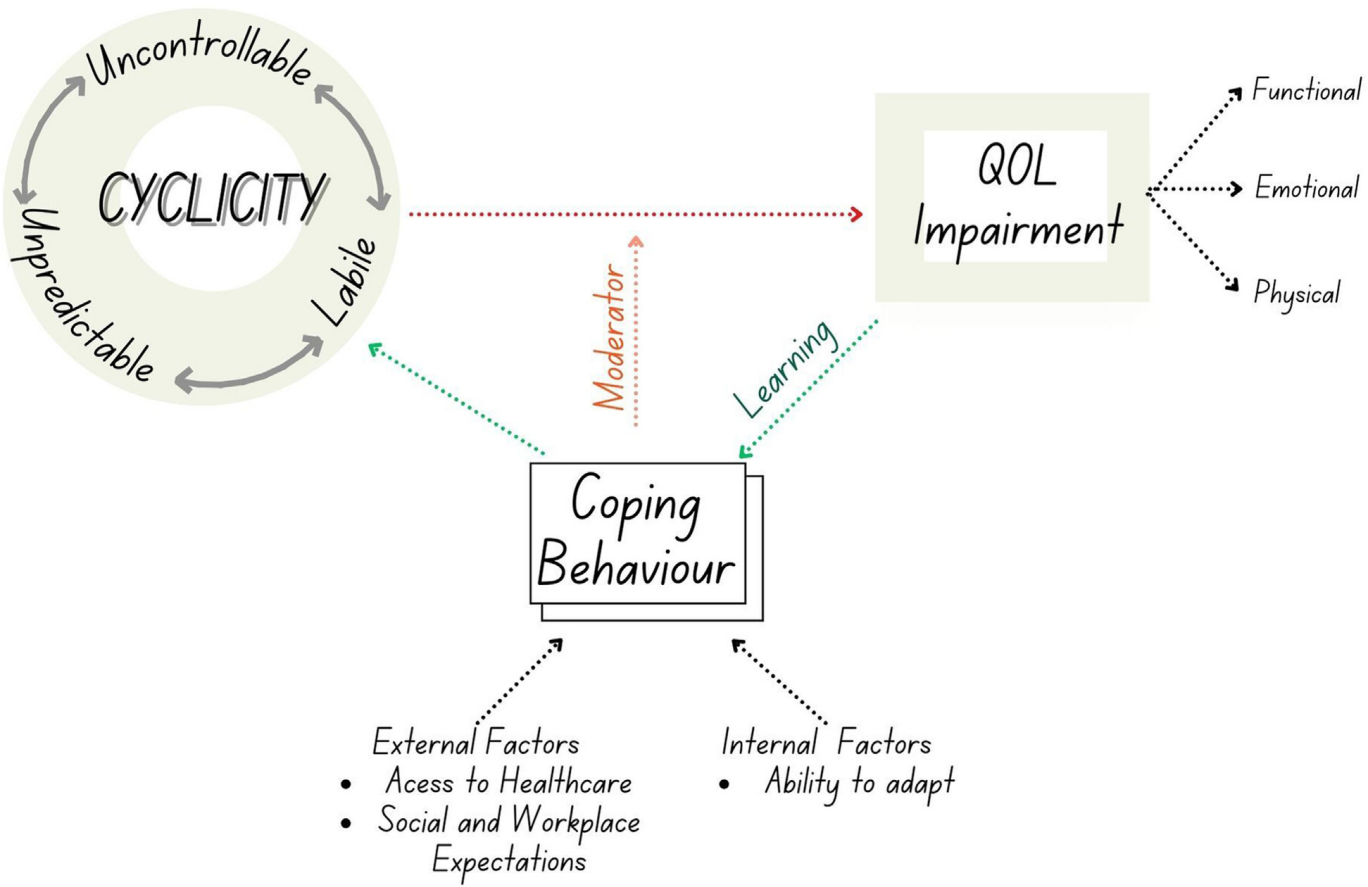
Relationship between disease cyclicity and QoL. *QoL*, Quality of life.

The derived multilevel multidimensional model (Fig 1) explains the relationship between cyclical conditions and QoL impairment. Based on the data, we generalize this model across various chronic dermatological conditions. At the individual level of analysis, a cyclical condition, defined by alternating periods of remission and activity, is characterized by predictability, controllability, and lability. Disease cyclicality contributes to the patient's variable and inconsistent ability to engage in daily activities that fluctuate between days of productivity and inactivity. This inconsistent productivity may be discordant with the demands at work and in social settings, thus impacting on QoL.

QoL impairment is moderated by internal factors such as the individual’s ability to adapt and community level-of-analysis factors such as social support and expectations from external parties. The ongoing experience and symptoms create a feedback loop of learning that may strengthen or weaken coping behaviors. These coping behaviors, such as the better management of triggers and early intervention during flares, can help mitigate the effects of cyclicity.

### Themes

The analysis revealed four main themes: (1) characteristics of a cyclical disease, (2) coping behaviors, (3) inconsistent ability, and (4) learning. These teams are further broken down into subthemes as illustrated in Table II and elaborated in Supplementary File 4, available via Mendeley at https://data.mendeley.com/datasets/r2dw9h9c2p/1.

**Table II:** Table of themes and representative quotes on how disease cyclicity impacts on quality of life

Theme	Representative quote
1. Characteristics of a cyclical disease	
1a. Predictability	
Timing of flares	‘Sometimes it really catches you off guard.’ ‘The unpredictability of it means that…I can never truly know when I'm going to have the onset of a flare.’ ‘The difficulty in predicting when it gets worse or when it gets better…that is the most difficult about this whole fluctuation.’
Severity of flare	‘Sometimes [it’s] a lot worse, sometimes it's not so bad…there's always an element of not knowing how bad the flare will be.’ ‘It can be really very painful or it can be very mild so it really depends on how bad the flare up is.’
1b. Controllability	
Lack of control of flares	‘I cannot control the highs and lows of it.’ ‘It's frustrating when you're already on the kind of gold standard therapy, and then you still have breakthrough symptoms.’ ‘Nothing can stop it from coming.’ ‘You can do all that you possibly can in your own strength to maintain at its best state. But … It could be anything that makes it suddenly worse… And you won't know what it is.’
1c. Labile (e.g. ability to change rapidly/quickly)	
Physical lability	‘When it gets worse, it's very steep, because it just happens overnight.’ ‘If you're not able to break the itch-scratch cycle between the second or third day, then your eczema…will just start to spiral down.’ ‘The most confusing part of managing it … I can do something. And suddenly it just gets better and I don't know why. And I'm like …I must be doing something right, and I continue with it. And all of a sudden it gets worse … this is something that I find most confusing and most difficult.’
Emotional lability	‘You’ll be happy when things are good. But when you get flares, then that kind of affects your mood. You get a bit more sad…a bit more anxious.’ ‘If it gets bad, I will be definitely annoyed for quite some time. My mood definitely [swings] below, down to a bad mood.’ ‘If you can't plan …what was initially a very good event, like has to be cancelled or has to be attended with great unhappiness or discomfort’

2. Coping behaviour - Internal factors	
2a. Ability to adapt	
Adapting physically	‘My body has a degree of pain sensitization because it's been experiencing cyclical pain for quite a while’ ‘I have adapted pretty ok with having it for so long...I don’t feel the heat even when it’s a normal heat.’ ‘I have lived there for so long… I don't mind the really bad pain… It just becomes a part of life….’ ‘I'm more used to … living with the itch. ….compared to some of my other friends who had eczema when they are adults, they seem to be bothered by the symptoms a lot more.’
Adapting functionally	‘I've made career choices to avoid certain situations that would be likely to exacerbate my symptoms.’ ‘When I have my normal session, without flares, I try to do things that I can do.’
Adapting emotionally	‘The long-term nature of flare ups…[trained] me to suppress those feelings of frustration. The discomfort is still there…but I just become better at redirecting my attention to other feelings or thoughts.’ ‘I think eventually I just got used to the frustration … knowing that it's in a cyclical manner, it will also get better… There's that hope.’ ‘We find our little ways of trying to manage the situation.’ ‘As I grew older, I became a bit more accepting… understanding of how the disease works … with that I didn't have that much... Self-blame or feelings of guilt.’

2. Coping behaviour - External factors	
2a. Social and workplace expectations	
Expectations at work	‘[Colleagues] will be wondering like ‘Why yesterday you can do this but today, you can't do this?’... They don’t get it that yesterday I was okay… but today, I’m not okay.’ ‘Your bosses or your teammates will expect you to perform at a consistent level, regardless of whether you're having a flaring disease or not.’
Expectations in social situations	‘[People who know me] know about my condition, and are quite polite about it, versus people who are new to me, they're like, ‘Oh, what's up with this guy?’ ‘New colleagues…ask [me] out for drinks… Sometimes they will be like, ‘what's up with this guy, he's not very sociable’... [Those] who know me well enough, they know that it's not because I don't want to.’
2b. Access to health care	
Treatment inconvenience	‘In order to get like certain types of creams, it needs to be prescribed, you can't just buy it off the shelf, you can't buy it anywhere.’
Scheduling difficulty	‘Whenever it flares up, the doctor has no time… The [consultation] will be at least 2 months after, it's like [there’s] no point.’ ‘It’s not like I can just say, ‘I have a very bad flare’ and I can just walk in… [By the time I see a dermatologist], the symptoms are gone.’
2c. Social support	
Social support	‘My boyfriend, he’s very used to it…when I flare up, then he’ll be the one helping me do the chores.’ ‘My family and my closest friends know about my condition…They would try and help if I need to take extra steps.’

3. Inconsistent ability	
Inconsistent ability	‘[When] my skin is much healthier, I feel more confident, I can go back and do more…. [but when] my eczema gets bad flare ups and stuff, it's very hard for me to go out and do stuff.’ ‘It's very hard to manage your life… let's say you're doing 1 year [of] studies… For 3 months, your skin is perfect, for [the following] 3 months, and your skin is like crap, then you may have to actually drop out [of studies] …’ ‘Mood becomes quite bad, like you just have low self-esteem and you just don't want to kind of meet people.’

#### Characteristics of a cyclical condition.

a. Predictability

Majority of the participants reported timing of their flares to be unpredictable that “really catches” them “off guard.” Others also found that flares can vary in severity, where “there's always an element of not knowing how bad the flare will be.” The inability to predict when symptom exacerbation would occur and the severity of each flare created challenges with making plans and disrupted daily routines.

b. Controllability

Many participants also described their cyclical flares as uncontrollable, where “nothing can stop it from coming.” They found that the inability to control the triggers or to abort the impending flare created a sense of loss of control, which was “frustrating” to many.

c. Lability

The rapid worsening and unpredictable nature of symptoms, where “when it gets worse, it’s very steep” or “it just happens overnight” was described by many participants. Some participants also reported emotional lability, where they found themselves shifting between emotions consisting of irritability, low mood, and self-esteem during flares.

**Fig 2:**
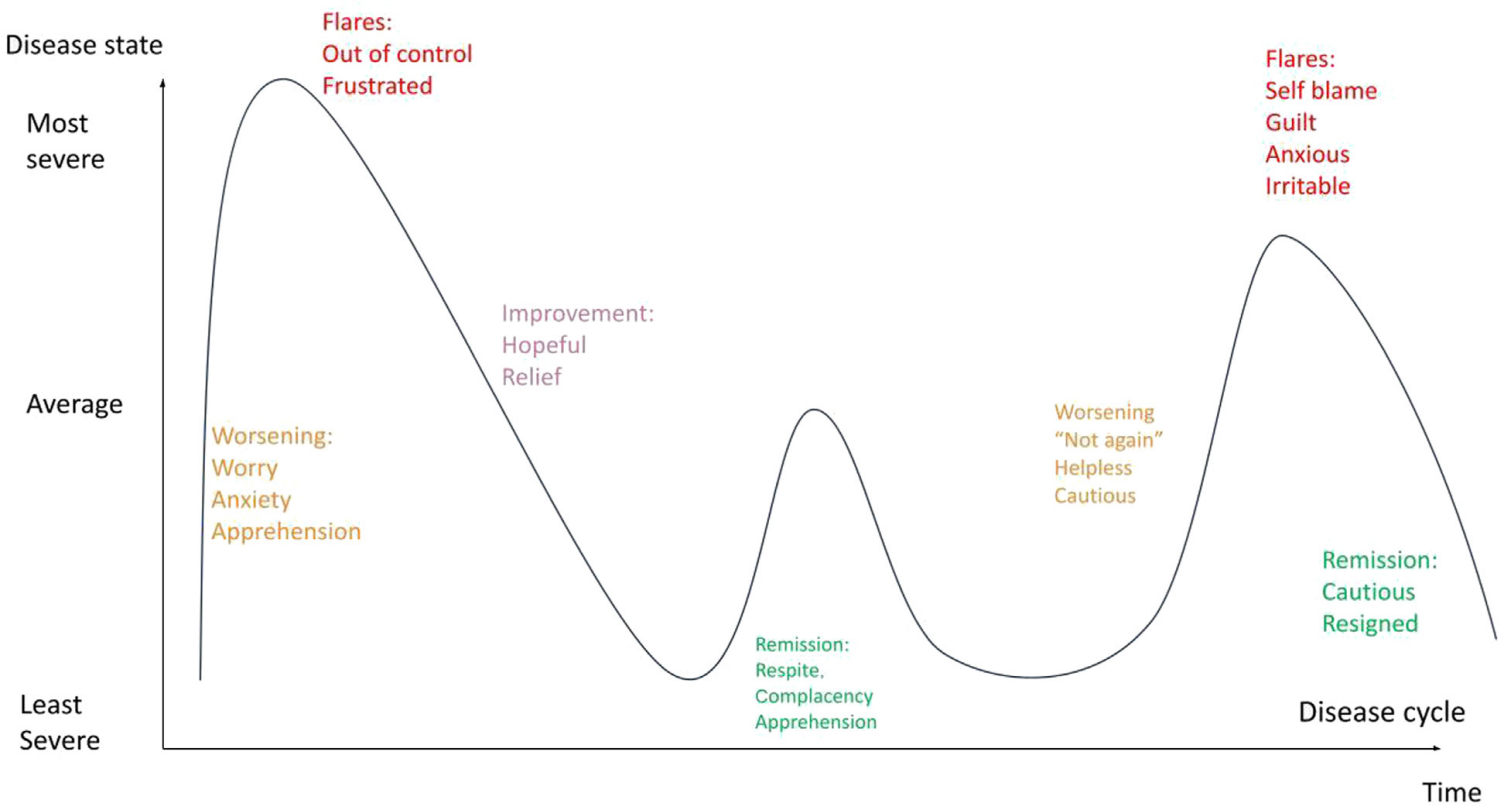
Phases of a cycle and the emotions experienced during each phase.

Fig 2 demonstrates the phases of a cycle, where each flare is unpredictable and does not follow a fixed temporal trajectory. An impending flare was associated with anxiety and apprehension where “you know, it's coming and nothing can stop it from coming.” Frustration over the lack of control of symptoms and flares was a common theme. During periods of improvement, many reported feeling “hopeful” with some becoming “complacent” and to “just enjoy when not having a flare.” With repeated flares, many felt “helpless” and “irritable” and some reported self-blame, “even though I’ve tried my best, my skin condition hasn’t improved.”

#### Coping behaviors

The impact of cyclicity on QoL is moderated by internal and external factors.

##### Internal factors:

a. Adapting physically, functionally, emotionally

Although many patients had skin symptoms that persisted over time, “the discomfort is still there, not reduced in any way.” However, they physically adapted by getting “used to…living with the itch.” “Itch… flakiness... fissures... to me it's normal to be that all the time.”

Emotionally adaptive patients grew more “open, accepting, and understanding” of their conditions and had less “self-blame or feelings of guilt” over time, choosing not to “let this stop me from living my life.” Functional adaptation was seen, for example by making “career choices to avoid certain situations,” or bringing a towel to sit on in order not to “leave skin flakes on the table or chair.”

The process of adapting to the symptoms and to the cyclical nature of disease was also seen in the interviews. These included maximizing productivity during remission, emotionally accepting the disease's cyclical nature, and learning to redirect negative emotions, “[The] long term nature of flare ups… train me to suppress those feelings of frustration… I just become better at redirecting my attention to other feelings.” Some challenges were evident in the adaptation process, such as, “[it was] confusing, do I adapt to the better baseline or… to the worst baseline?”

##### External factors:

a. Social and workplace expectations:

While disease cyclicity caused individual productivity to be inconsistent, the workplace demands remained constant, causing a dissonance between the individual and the work group, “Your bosses or your teammates will expect you to perform at a consistent level, regardless of whether you're having a flaring disease.” These external demands were moderated with better understanding and strong social support.

b. Access to health care:

The unpredictable nature of disease cyclicity makes it difficult to access timely health care. One patient mentioned, “the doctor has no time… the meeting will be at least like 2 months after (the request for an appointment).” The need for prescriptive medications and adjustments to the treatment regime also created treatment inconvenience when those needs were not met in a timely manner.

c. Social support

Empathy and support from family, friends, and colleagues moderate the relationship between disease cyclicity and QoL impairment. Some are “very understanding” and made allowances during periods of flares, while others are unsupportive, leading to “a lot of emotional and psychological stress.”

#### Inconsistent ability

Inconsistent ability refers to the variable and fluctuating nature of the person ability to perform daily activities whether at work or in a social setting. This arises because during disease flares when symptoms were exacerbated, participants experienced a reduction in physical form, cognitive state and energy level. This negatively affected their work activities like “meeting clients” and family commitments, “if my kid wants to go out to the beach… I can only observe her from the side.” Social activities were also limited during disease flares and “it is very hard for me to go out and do stuff and spend time with people as well.”

#### Learning

The experience and challenges of a cyclical and chronic condition created an opportunity for learning and adaptation, where coping behaviors may be strengthened, and patients found their “little ways of trying to manage.” For example, to the common complaint of unsolicited advice from friends and family, a patient jokingly shared, “I think I've adapted over time, they get less irritating.”

## Discussion

The results emphasize the importance of looking beyond just the objective physical state of disease. Independent of objective severity, a condition that is unpredictable, uncontrollable, and labile hinders a person’s ability to perform daily tasks consistently and makes it difficult for the person to adjust and adapt to a new normal.^4^^,^^10^ The burden of symptoms is further influenced by the interaction between the individual and her environment.

To contextualize our findings, we refer to the Transactional Model of Stress and Coping^11^ by Lazarus, which describes how stress and coping is a dynamic interaction between a person’s interpretation of the stressor and the resources for coping such as knowledge, health, and social support. Coping strategies can be divided into problem-focused and emotion-focused coping. Similarities in both models include the dynamic nature of internal and external factors with constant reappraisal. While the Transactional Model of Stress of Coping focuses heavily on how the individual’s cognitive processes influence coping, our model emphasizes understanding the relationship of disease characteristics and QoL impairment outcomes. This approach is likely to be more useful in understanding coping behaviors during illness and disease.

In the context of these findings, we propose four practical implications to mitigate the impact of disease cyclicity and QoL. First, biomedical therapeutics that produce a stable but suboptimal disease state may be more beneficial than those targeting immediate improvements such as disease remission that do not last very long.

Second, individual adaptation should occur for symptoms (eg, skin itching, avoidance of triggers) and the fluctuating nature of disease. The latter involves emotionally accepting cyclicity, being flexible in planning daily tasks, and establishing contingencies that respond to unpredictable disease fluctuations. Our interviews reflect that patients also do adapt and “get used” to the recurrent flares of their conditions.

Third, we need to bridge the discordance between the individual’s shifting ability to perform daily tasks and the expectations and demands from external parties. This may involve a patient being open to discuss her challenges with relevant external stakeholders (employees, family members, friend networks, etc.), combined with a deeper level of understanding from the stakeholder community. Finally, access to timely care during flares is a critical hygiene factor for effectively managing the disease. However, with the widespread shortage of health care providers and rigidity of health care payment systems calls for innovations in service delivery models, home care, telemedicine, empowering provider proxies such as nurse practitioners and community pharmacists. In short, there is a need to expand service models that enable flexible access to care during flares.^12^^,^^13^

Strengths of the study include the comprehensive and synergistic use of qualitative and quantitative methods. The inclusion of nondermatological conditions broadens the applicability of our theory. Limitations of the study include the assumption that participants have accurate insights into their own behaviors, which may not necessarily be true. Additionally, given the cultural relevance of symptom burden and societal norms, these results may not be generalizable to other cultures or populations. The study's scope was also confined to a limited number of dermatological and rheumatological conditions. Future research is needed to delve deeper into the development of targeted interventions such as education programs that address the unique challenges posed by the cyclical nature of dermatological conditions. A broader spectrum of cyclical diseases across various disciplines can also be explored.

## Conflicts of interest

None disclosed.
